# Nobiletin restores the intestinal barrier of HFD-induced obese mice by promoting MHC-II expression and lipid metabolism

**DOI:** 10.1186/s10020-025-01072-1

**Published:** 2025-01-26

**Authors:** Ni Yang, Yue-Shan Pang, Yali Zheng, Yan-Ju Gong, Wei-Jun Ding

**Affiliations:** Department of Fundamental Medicine, Chengdu University of Traditional Chinese Medicine, Chengdu, 610072 Sichuan Province China

**Keywords:** High-fat diet (HFD), Nobiletin, Intestinal barrier, Major histocompatibility complex class-II (MHC-II), Lipid metabolism

## Abstract

The incidence of obesity is increasing annually worldwide. A high-fat diet (HFD) causes intestinal barrier damage, but effective interventions are currently unavailable. Our previous work demonstrated the therapeutic effect of nobiletin on obese mice; thus, we hypothesized that nobiletin could reverse HFD-induced damage to the intestinal barrier. Male C57BL/6 J mice were orally administered nobiletin for 14 d. After identification, the obese mice were equally divided into three groups: the HFD group, the low-dose (NOL, 100 mg/kg/d) group and the high-dose nobiletin (NOH, 200 mg/kg/d) group. A normal control group (CON) was also included. Hematoxylin and eosin (HE) staining and immunofluorescence were used to observe the intestinal barrier. RT-qPCR was used to determine the transcriptomic levels of genes involved in intestinal barrier integrity and lipid metabolism. The results revealed that intestinal tight proteins, including ZO-1 and Occludin, were significantly reduced in HFD-fed mice but markedly restored after nobiletin intervention, particularly in NOH mice. Improvements in the intestinal barrier and lipid metabolism associated with major histocompatibility complex class II (MHC-II) and relevant elements were revealed after nobiletin intervention. Enrichment analysis revealed that MHC-II plays an important role in the restoration of the intestinal barrier. Taken together, nobiletin restored intestinal barrier integrity and lipid metabolism by regulating MHC-II expression.

## Induction

Obesity is a globally increasing disease that affects billons of people (Haczeyni et al. 2018) and is caused by multiple factors, particularly abnormal diets (Turner-McGrievy and Grant 2015). A high-fat diet (HFD) can promote intestinal inflammation (Kreuter et al. 2019), increase serum TNF-α and IL-6 levels (Schmid et al. 2015) and downregulate tight junction proteins (Poritz et al. 2007). A HFD directly impairs the intestinal barrier, which in turn increases intestinal permeability and leads to leaky gut (Rohr et al. 2020). Animal studies have shown that a HFD induces or exacerbates intestinal inflammation and decreases the expression of genes associated with tight junction proteins in epithelial cells, thereby increasing intestinal permeability (Cani et al. 2008). A HFD also leads to low-grade inflammation and an impaired intestinal barrier in zebrafish (Arias-Jayo et al. 2018).

Nobiletin, a flavonoid isolated from the peel of citrus fruits (Huang et al. 2016) has been shown to have multiple pharmaceutical effects, including anti-inflammatory, immunomodulatory, anticancer, antioxidative, neuroprotective and antiatherosclerotic effects (Arias-Jayo et al. 2018; Huang et al. 2016). It was reported to attenuate dextrose sodium sulfate-induced intestinal barrier damage through modulating the HNF4α-Claudin-7 signaling pathway (Wen et al. 2020), ameliorating enteric inflammation (Li et al. 2022) and restoring intestinal barrier function (Xiong et al. 2015). In inflammatory bowel disease, nobiletin reduces the expression of inflammatory symptoms and markers for chronic colitis (Hagenlocher et al. 2019). Nobiletin ameliorated experimental colitis by reducing inflammation and restoring impaired intestinal barrier function (Xiong et al. 2015; He et al. 2019) and restored antibiotic-induced intestinal barrier dysfunction by upregulating intestinal tight junction protein expression (Zhan et al. 2024). Zhao et al. reported that nobiletin inhibited lipid metabolism-related genes, reversed HFD-induced dysbiosis of the gut microbiota, and improved intestinal diversity in HFD-fed mice (Zhao et al. 2023). These results suggest that nobiletin improves lipid metabolism and inflammation in obese mice (Pang et al. 2022).

However, no publications have focused on the restoration of HFD-induced intestinal barrier dysfunction with nobiletin. Herein, we generated HFD-induced obese mice, revealed the anti-inflammatory effects and restoration of the intestinal barrier by nobiletin, and primarily probed the underlying mechanism involved.

## Materials and methods

### Animals and nobiletin administration

All animal experiments were performed according to the protocol approved by the Ethics Committee of Chengdu University of Traditional Chinese Medicine (License: 2021-05). Ninety 8-week-old male C57BL/6 mice were purchased from Spafford; 10 of these mice were fed low-fat chow (D12450J, USA) as a normal control group, and the remaining mice were fed high-fat chow (D12492, USA). The obese mice were identified according to our previous publication (Xia et al. 2022) and randomly grouped for further intervention. The nobiletin (Ruifensi Biotech Co., Ltd., Chengdu, China) (purity > 98%) concentration was determined according to previous methods (Pang et al. 2022). In the early stages of the experiment, a dose of 40 mg/kg/d was used, but the results were the same as those for 100 mg/kg/d, so the 40 mg/kg/d was omitted in the writing. Low- (NOL, 100 mg/kg/d) and high-dose nobiletin (NOH, 200 mg/kg/d) mice were treated for 2 w. Equal volumes of PBS were used in the CON and HFD groups.

### Determination of the obesity index

Body weights were measured and recorded weekly. The body length of the mice was measured at the end of the experiment, and Lee’s index was calculated. An oral glucose tolerance test (OGTT) was simultaneously performed.

### Serum and tissue sample collection

The mice were anesthetized with isoflurane (RWD Life Sciences, Shenzhen, China), and approximately 500 μl of serum sample was collected from each subject. Serum levels of high-density lipoprotein (HDL), low-density lipoprotein (LDL), total cholesterol (TC) and triglyceride (TG) were assayed using an automated biochemical analyzer (BS-240VET, Myriad Co., Shenzhen, China). The assay kits were purchased from Myriad Corporation (Shenzhen, China). The small intestine, liver and epididymis were anatomically removed and fixed with 4% paraformaldehyde. The remaining small intestine samples were quickly frozen in liquid nitrogen and stored at − 80 °C.

### Hematoxylin–eosin (HE) staining and immunofluorescence

HE staining was performed via a conventional approach. Samples of small intestinal tissues were embedded in paraffin. After dewaxing, the 5 μm sections were stained with hematoxylin and eosin, dehydrated and sealed. Photographs were obtained by a light microscope (Leica, Italy). The images were analyzed with ImageJ.

The above tissue sections were further subjected to immunofluorescence staining. The sections were first deparaffinized with xylene and ethanol and then antigenically repaired with EDTA. Bovine serum was added for 10 min at room temperature, and the samples were incubated with anti-ZO-1/Occludin antibodies for 1 h. The samples were subsequently washed five times, incubated with AlexaFluor^®^488 (ab150129) and AlexaFluor^®^647 in a dark environment for 1 h, and incubated with DAPI for 1 h. Images were taken with a fluorescence microscope (Leica, Italy). Four fields of view were collected from the upper, lower, left and right parts of each section. The optical density was quantified and determined using ImageJ software.

### RT-qPCR

Total RNA was extracted from small intestinal tissues using an RNA extraction kit (Forgene, China) and reverse transcribed with a SuperMix (Vazyme Biotechnology Co., Ltd., Nanjing, China). cDNA libraries were constructed on a PROMETHION platform (Biomarker, Inc., Beijing, China). The expression levels of the genes were determined via real-time PCR (Vazyme Biotechnology Co., Ltd., Nanjing, China). The *β-actin* gene was used as an internal control (Table 1). The 2^−∆∆Ct^ method was used to calculate the fold change.

**Table 1:** Sequences of the primers used for RT-qPCR

Gene name	Forword primer	Reword primer
*Pla2g5*	5′-TGCTGTCAGATGCACGACC	5′-TTCGCAGATGACTAGGCCATT
*Pla2g2f*	5′-GCCTCTCCCTCTAAAACCTCC	5′-AGCACCAGTCTACCTCATCCA
*H2-DMb2*	5′-GGAGGGGGTTCAAGGTCTTC	5′-AGCCAGGAATTCCACCAGTTTA
*Abca1*	5′-GCTTGTTGGCCTCAGTTAGG	5′-GTAGCTCAGGCGTACAGAGAT
*Cd8b1*	5′-CTCTGGCTGGTCTTCAGTATA	5′-TCTTTGCCGTATGGTTGGTTT
*Apoa4*	5′-CCAATGTGGTGTGGGATTACTT	5′-AGTGACATCCGTCTTCTGAAAC
*Acaa1a*	5′-ACGCATCGCCCAATTTCTGA	5′-CCAGACAGGGACATGGACTC
*Acadl*	5′-TTTCCTCGGAGCATGACATTT	5′-GCCAGCTTTTTCCCAGACCT
*Scd2*	5′-GATCTCTGGCGCTTACTCAGC	5′-CTCCCCAGTGGTGAGAACTC
*IL-17*	5′-CAGCTCAGTAACAGTCCGCC	5′-TCTCGACCCTAAAGTGAA
*ZO-1*	5′-GCCGCTAAGAGCACAGCAA	5′-GCCCTCCTTTTAACACATCAGA
*Occludin 1*	5′-TGAAAGTCCACCTCCTTACAGA	5′-CCGGATAAAAAGAGTACGCTGG
*IL-22*	5′-ATGAGTTTTTCCCTTATGGGGAC	5′-GCTGGAAGTTGGACACCTCAA

### Full-length transcriptomic sequencing

Differences of interest (*p* < 0.05, |fold change|> 1.2) were considered significant. Volcano maps of the differentially expressed genes (DEGs) were generated using the DESeq package (1.18.0) (https://cloud.oebiotech.cn). Kyoto Encyclopedia of Genes and Genomes (KEGG) and Gene Ontology (GO) enrichment analyses were performed (https://Metascape.org/gp/), and the results are plotted in http://www.bioinformatics.com.

### Statistics

Statistical analysis and plotting were performed using GraphPad Prism (V9.1.0). Normally distributed data are expressed as the means ± SDs. Statistical analysis was performed via one-way or two-way ANOVA. All tests were considered to be statistically significant at *p* < 0.05.

## Results

### Nobiletin ameliorated obesity

**Fig. 1:**
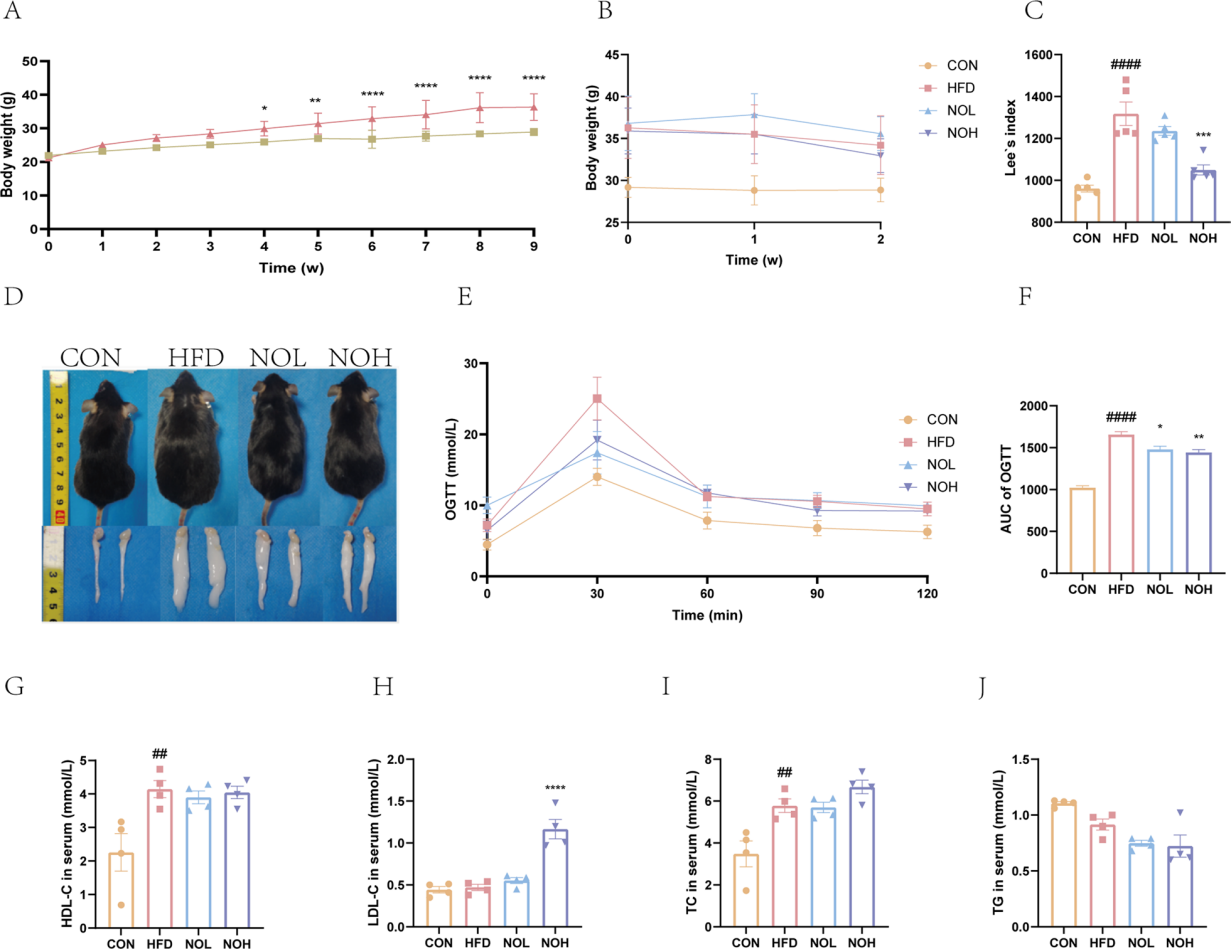
Nobiletin ameliorated HFD-induced obesity. **a**. Time series of the body weights. **b**. Body weight changes after nobiletin intervention. **c**. Lee’s index was determined at the end of the experiment. **d**. Representative body size and morphology of epididymal lipid samples. The OGTT results (**e**) and the area under the curve (AUC) of the OGTT (**f**). Serum levels of HDL-C (**g**), LDL-C (**h**), TC (**i**) and TG (**j**). Compared with those in the CON group, ^####^*p* < 0.0001, ^##^*p* < 0.01, and ^#^*p* < 0.05. Compared with those of the HFD group, ****p < 0.0001, ***p < 0.001, **p < 0.01, and *p < 0.05. OGTT, oral glucose tolerance test; HFD, high-fat diet group; CON, normal group; NOL, low-dose nobiletin; NOH, high-dose nobiletin

Significantly increased body weights were observed after 4 weeks of HFD consumption (Fig. 1a). Compared with those in the HFD group, the average body weights of the NOL and NOH groups were substantially lower although the difference was not significant (Fig. 1b). Nobiletin also markedly reduced Lee’s index (Fig. 1c) and the amount of epididymal fat (Fig. 1d). A HFD significantly increased the serum glucose concentration and induced glucose tolerance, but these effects were markedly ameliorated in the NOL and NOH groups (Fig. 1e, f). The levels of key obesity indices were significantly increased in obese mice (Fig. 1g, i). Interestingly, the serum LDL-C levels were significantly increased after NOH intervention (Fig. 1h).

### Nobiletin inhibited lipid accumulation and reversed HFD-induced damage

**Fig. 2:**
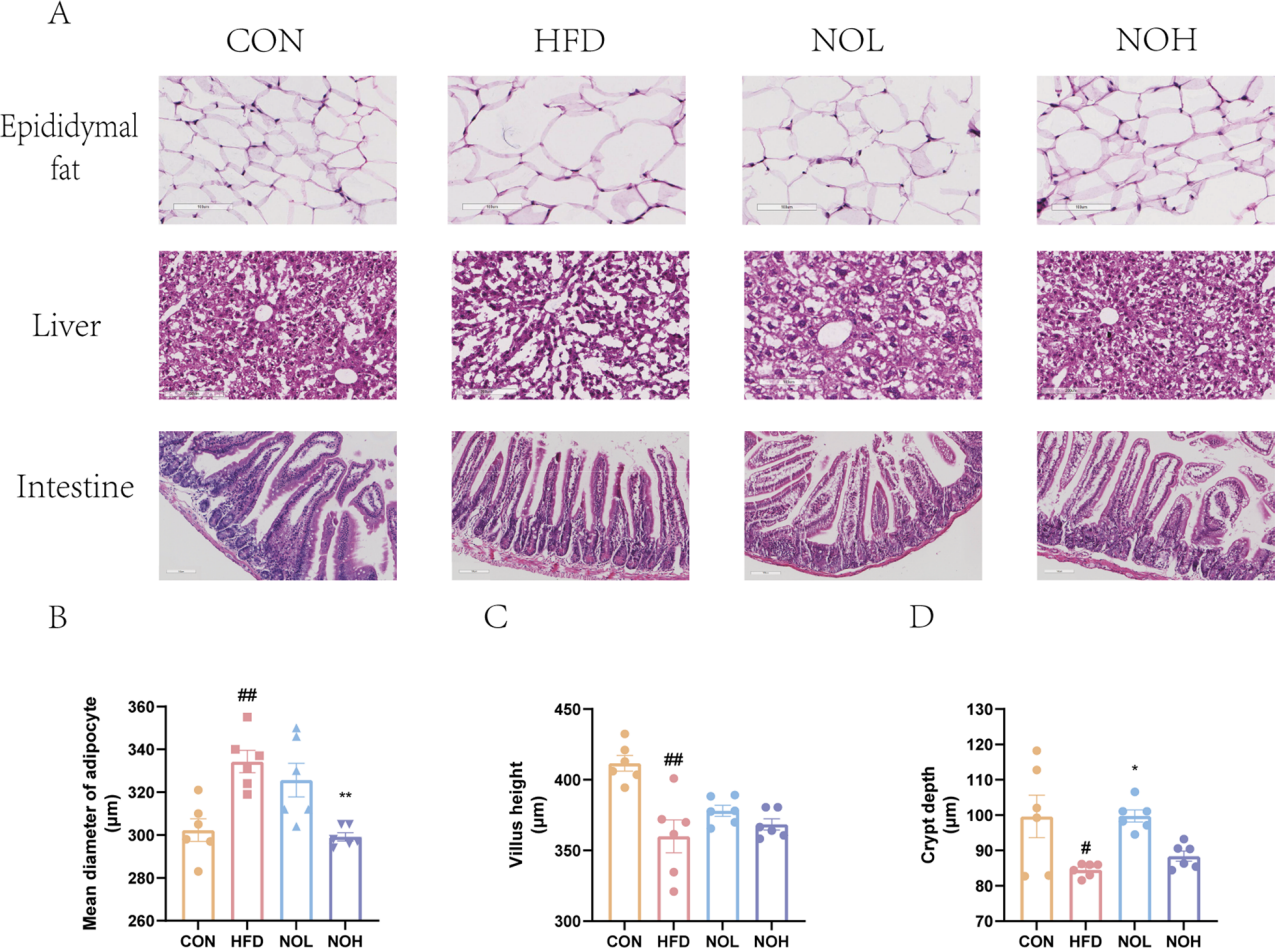
Nobiletin inhibited lipid accumulation and restored HFD-induced pathological alterations. **a**. HE staining. **b**. Average diameter of adipocytes. The depth of the intestinal villi (**c**) and crypts (**d**). The results are presented as the means ± SDs. Compared with the CON group, ^##^*p* < 0.01, ^#^*p* < 0.05. Compared with the HFD group, **p < 0.01, *p < 0.05. HFD, high-fat diet group; CON, normal group; NOL, low-dose nobiletin; NOH, high-dose nobiletin

Notably, reduced lipid accumulation was observed in the epididymal fat, liver and intestine after nobiletin intervention (Fig. 2a). In particular, the intestinal lipids in the NOH group almost reached the level observed in the CON group (Fig. 2a). Additionally, the average diameter of adipocytes and the depth of crypts were significantly lower in the NOH group than in the HFD or CON group (Fig. 2b–d).

### Nobiletin restored HFD-induced injury to the intestinal barrier

**Fig. 3:**
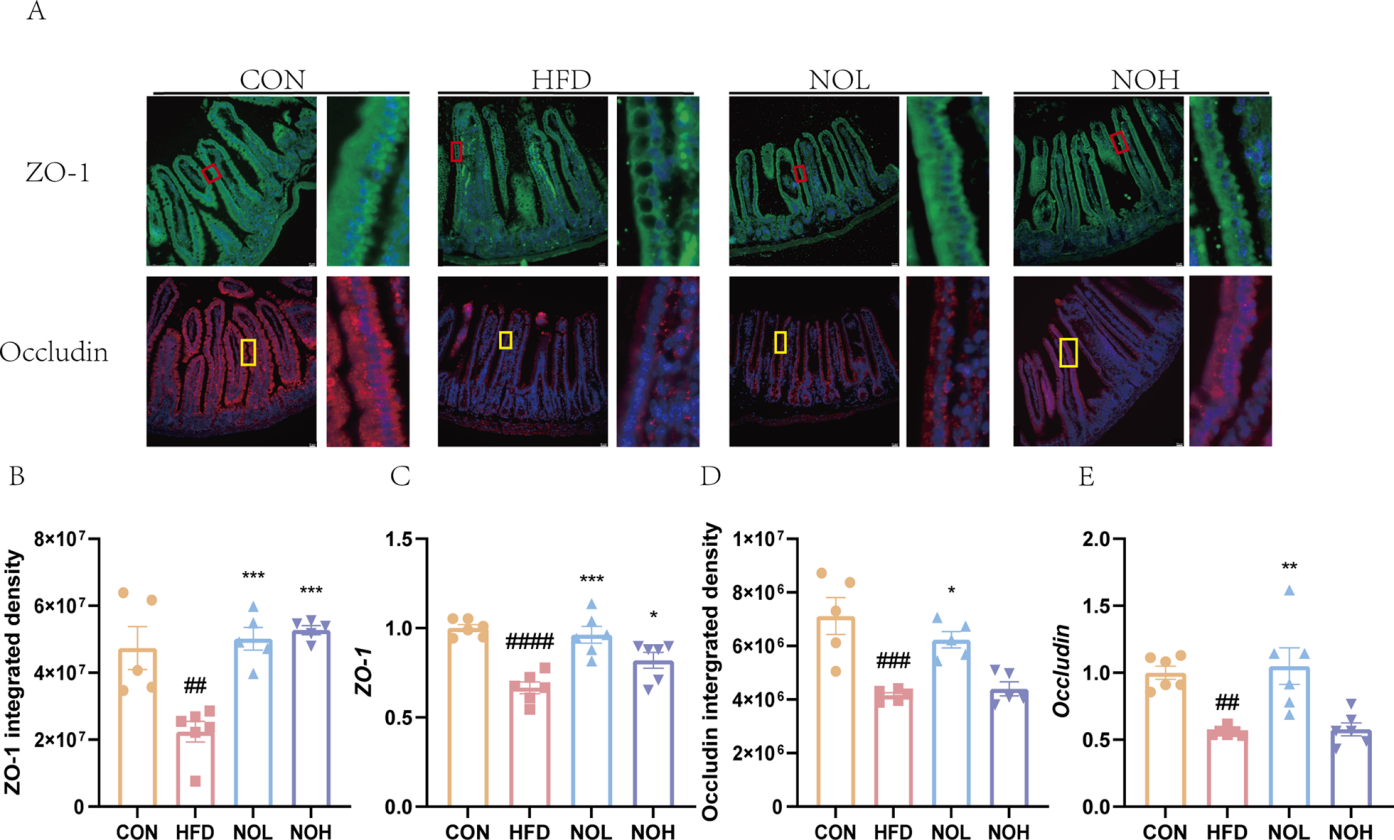
Nobiletin restored HFD-induced damage to the small intestinal barrier. **a**. Representative images of ZO-1 and Occludin immunofluorescence. The average optical density of ZO-1 (**b**) and Occludin (**d**). Gene expression levels of *ZO-1* (**c**) and *Occludin* (**e**). Compared with those in the CON group, ^####^*p* < 0.0001, ^###^*p* < 0.001, and ^###^*p* < 0.01. Compared with the HFD group, ***p < 0.001, **p < 0.01, and *p < 0.05. HFD, high-fat diet group; CON, normal group; NOL, low-dose nobiletin; NOH, high-dose nobiletin

The intestinal proteins ZO-1 and Occludin are critical factors involved in enteric integration. The expression of both the ZO-1 and Occludin proteins and genes decreased in obese mice but was significantly restored after nobiletin intervention, especially in the NOH group (Fig. 3), suggesting that these intestinal barriers were effectively restored by nobiletin treatment.

### Nobiletin rescued HED-induced damage to the intestinal barrier by regulating the expression of key genes involved in MHC-II

**Fig. 4:**
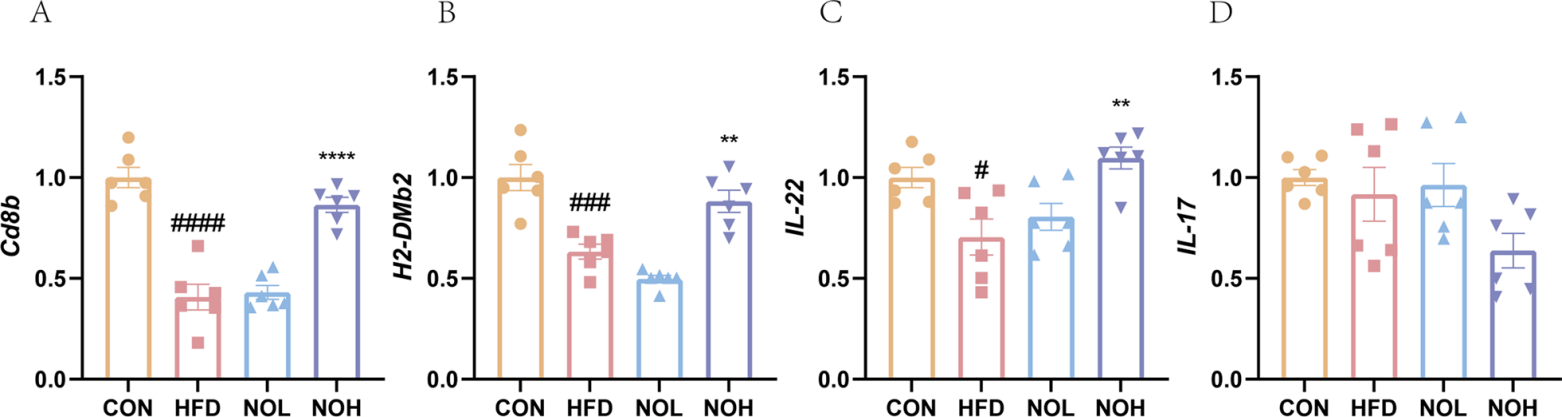
Nobiletin regulates the expression of key MHC-II genes associated with lipid metabolism. *Cd8b*, T-cell effector gene; *H2-DMb2*, major histocompatibility complex class II gene; *IL-22*, interleukin-22; *IL-17*, interleukin-17. The results are presented as the means ± SDs. ^####^*p* < 0.0001, ^###^*p* < 0.001, and ^#^*p* < 0.05 compared with the CON group. ****p < 0.0001, **p < 0.01 compared with the HFD group. HFD, high-fat diet group; CON, normal group; NOL, low-dose nobiletin; NOH, medium-dose nobiletin; NOH, high-dose nobiletin

The RT-qPCR results revealed that the expression levels of the *CD8B, H2-DMb2*, and *IL-22* genes were markedly decreased in the HFD group but significantly increased in the NOH group (Fig. 4). *H2-DMb2* is an *MHC-II* gene (Yang et al. 2021). These findings indicate a potential mechanism by which nobiletin protects against obesity-induced leaky gut tissue by effectively improving the transcriptomic activity of *MHC-II*.

### Nobiletin tunes the intestinal transcriptome to ameliorate obesity

**Fig. 5:**
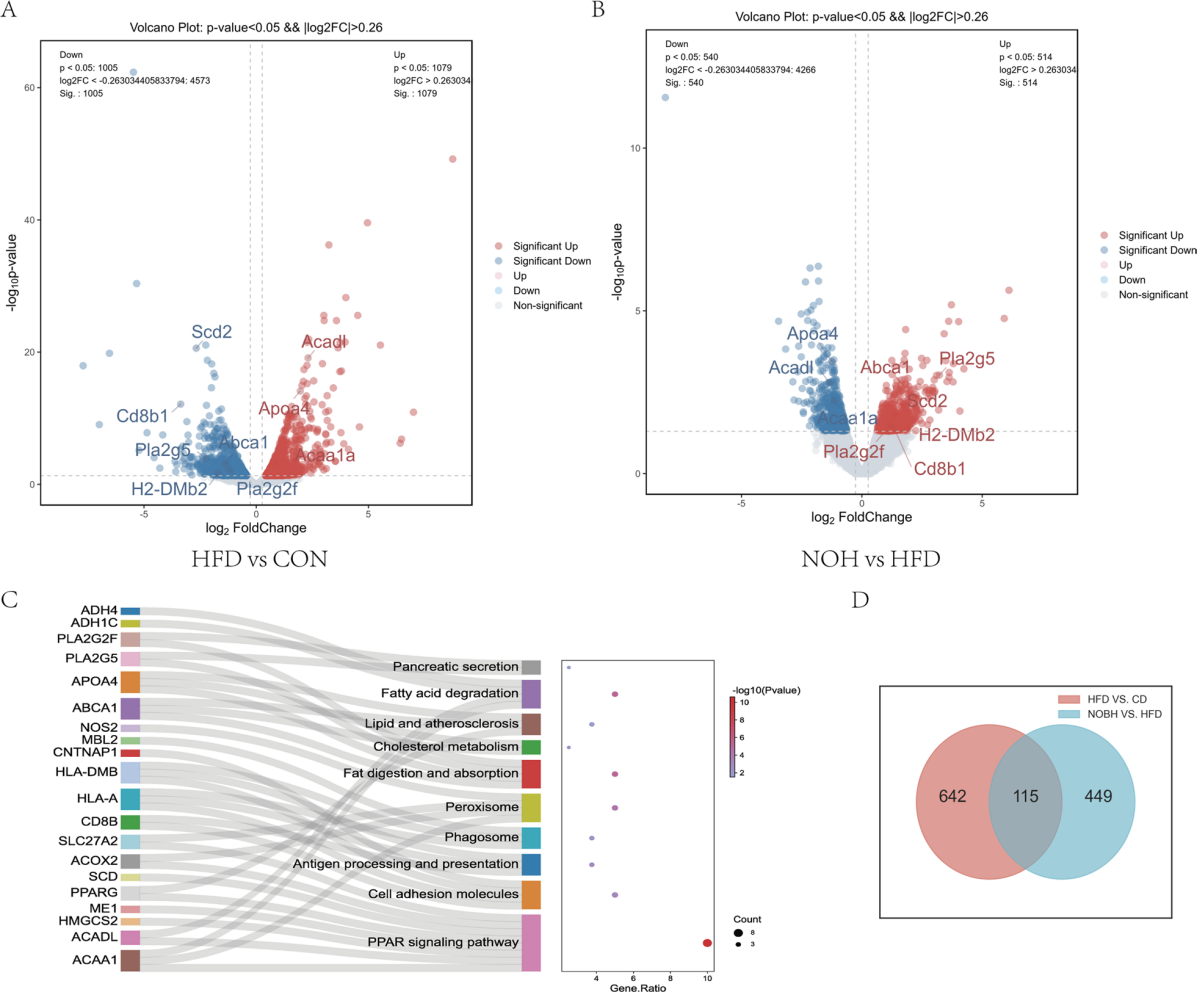
Transcriptomic regulation of the small intestine by nobiletin. **a** and **b**. Volcano plots. **c**. KEGG enrichment analysis. **d**. Wayne plots. HFD, high-fat diet group; CON, normal group; NOL, low-dose nobiletin; NOH, high-dose nobiletin

To further explore the underlying mechanism by which nobiletin protects against obesity, full-length transcriptome sequencing was performed on samples from the small intestines. A total of 757 DEGs were identified between the HFD and CON groups, and 564 DEGs were identified between the NOH and HFD groups (Fig. 5a, b). The volcano plots show 115 DEGs between the HFD and NOH groups, which are related mainly to lipid metabolism and immunomodulation, including *Cd8b* and *Acad1* (Fig. 5c, d).

**Fig. 6:**
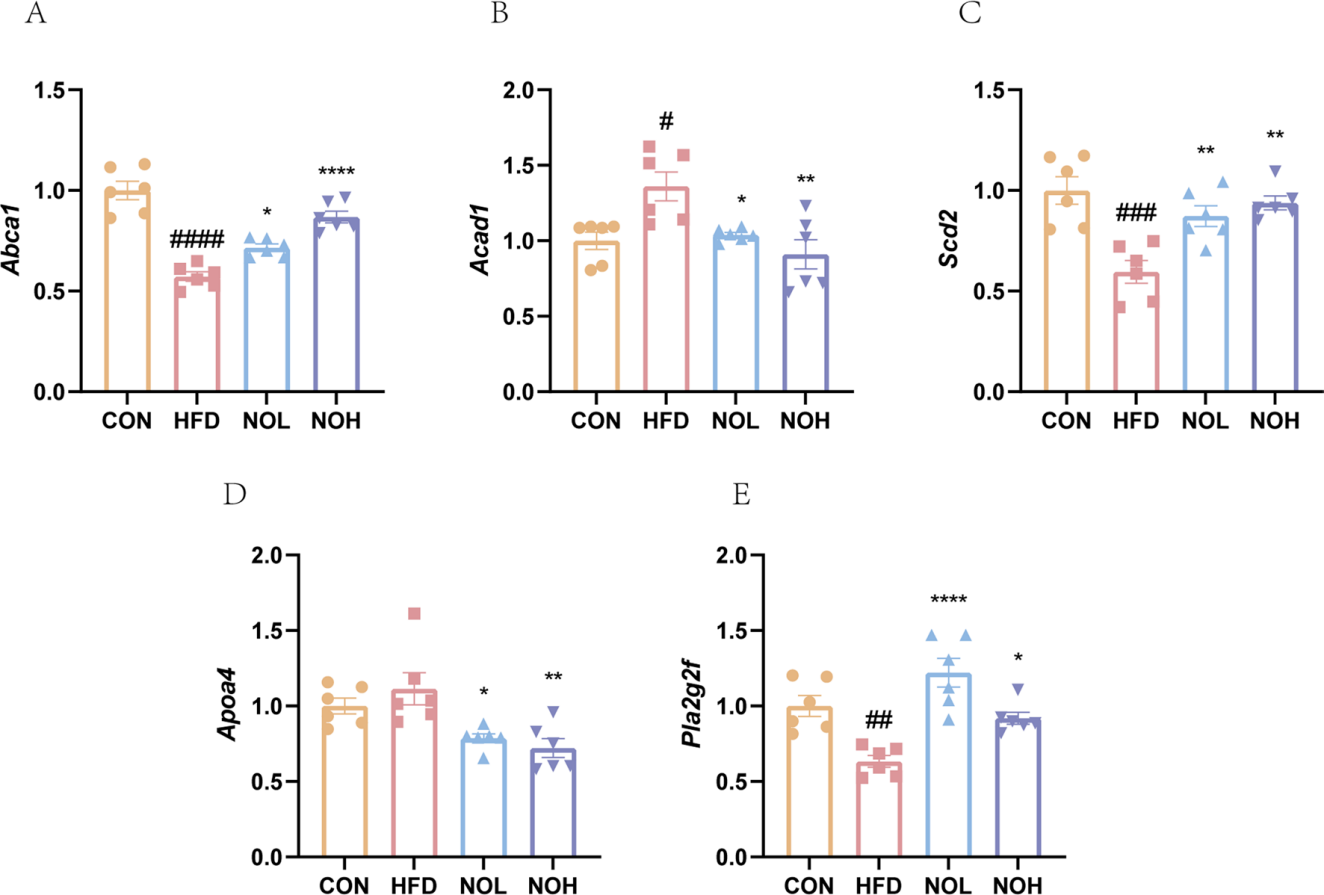
Nobiletin ameliorated obesity by regulating genes associated with lipid metabolism. The expression of the *Abca1* (**a**), *Acad1* (**b**), *Scd2* (**c**), *Apoa4* (**d**) and *Pla2g2f genes*. The results are presented as the means ± SDs. ^####^*p* < 0.0001, ^###^*p* < 0.001, ^###^*p* < 0.01 and ^#^*p* < 0.05, compared with the CON group. ****p < 0.0001, **p < 0.01 and *p < 0.05, compared with the HFD group

To confirm the antiobesity effects of nobiletin, key genes involved in lipid metabolism were evaluated via RT-qPCR. The results revealed that the *Abca1*, *Scd2* and *Pla2g2f* genes were upregulated after nobiletin intervention (Fig. 6a, c and e), whereas *Acad1* and *Apoa4* were downregulated (Fig. 6b–d).

## Discussion

Our work revealed that nobiletin extensively improved obesity indices including body weight, Lee’s index and hepatic lipid accumulation (Pang et al. 2022), particularly restoring the intestinal barrier in obese mice via the upregulation of MHC-II expression.

Our findings suggest that lipid metabolism-related gene expression is restored after nobiletin treatment. Lipid accumulation is significantly increased in the *Acadm* knockout mouse model (Ma et al. 2021). Significant downregulation of the Acad1 gene with disease progression was found in biopsy specimens from patients with nonalcoholic fatty liver disease (Mitsuyoshi et al. 2009). *Pla2g5f* is a linoleic acid and arachidonic acid metabolism gene (Vomhof-DeKrey et al. 2019). Scd catalyzes the conversion of saturated fatty acids into monounsaturated fatty acids, a key step involved in lipid metabolism (Zhang et al. 2005). Diets with saturated fatty acids promote proinflammatory effects in the gut (Basson et al. 2020). Shi et al. reported that saturated fatty acids lead to the release of proinflammatory factors, impairment of intestinal barrier function and disruption of cellular metabolism (Shi et al. 2006). Our work indicated that the ability of nobiletin to eliminate accumulated lipids in HFD mice is associated with injury to the intestinal barrier, while nobiletin restored the intestinal barrier by suppressing abnormal lipid accumulation.

The present work showed that both low and high doses of nobiletin can effectively restore intestinal barrier injury in obese mice. A HFD leads to a significant decrease in the expression of the barrier-forming cytokine IL-22 (Rohr et al. 2020), which results in altered intestinal permeability (ZO-1 and Occludin) (Serre et al. 2010). Nobiletin can attenuate dextran sodium sulfate-induced intestinal barrier damage via the HNF4α-Claudin-7 signaling pathway (Wen et al. 2020) and can cure experimental colitis by reducing inflammation and restoring the damaged intestinal barrier (Xiong et al. 2015). Nobiletin can also ameliorate antibiotic-induced intestinal barrier disorders and increase intestinal tight junction and progenitor cell numbers (Zhan et al. 2024). Resuming the damaged gut barrier might be a potential mechanism by which nobiletin protects against obesity.

Our study demonstrated that nobiletin enhances the intestinal barrier by increasing the levels of MHC-II and relevant proteins. The gut prevents substances such as harmful microorganisms, antigens, and toxins from invading mucosal tissues and ultimately the body circulation (Usuda et al. 2021). Therefore, the integrity of the intestinal barrier is rather important. There is growing evidence that maintenance of the intestinal barrier can be coordinated with intestinal epithelial cells through the intestinal immune system (Serre et al. 2010). A previous publication reported that MHC-II could be regulated by TLR2/Myd88 and IFNγ signaling in intestinal epithelial cells induced by gram-negative *Helicobacter pylori* species (Beyaz et al. 2021; Mandell et al. 2004). Our results revealed for the first time that a HFD induced a decrease in MHC-II expression (Rohr et al. 2020). MHC-II in IECs can capture, process and present antigens to CD4^+^ T cells (Biton et al. 2018), while CD4^+^ T cells produce IL-22 (Yang et al. 2020). Thus, MHC-II might play an important role in the intestinal barrier.

Overall, nobiletin promoted the release of cytokines from the small intestinal barrier and the restoration of the small intestinal barrier through MHC-II expression. These results suggest that nobiletin has a restorative effect on the intestinal barrier through its ability to improve intestinal tight junctions. Moreover, nobiletin improved lipid metabolism and decreased lipid accumulation, thereby reducing the degree of intestinal damage caused by lipid accumulation. This study describe the effects of nobiletin on HFD-induced intestinal barrier dysfunction.

## Conclusion

Nobiletin ameliorated intestinal barrier injury and excess lipid metabolism in obese mice, and MHC-II is a potential target of nobiletin.

## Data Availability

The authors confirm that the data and materials supporting the findings of this study are available within the article.

## Abbreviations

HFD: High-fat diet
NOL: Low-dose nobiletin group
NOH: High-dose nobiletin group
CON: Normal control group
HE: Hematoxylin and eosin
MHC-II: Major histocompatibility complex class II
OGTT: Oral glucose tolerance test
HDL: Serum levels of high-density lipoprotein
LDL: Low-density lipoprotein
TC: Total cholesterol
TG: Triglyceride
